# Automatic Regionalization of Model Parameters for Hydrological Models

**DOI:** 10.1029/2022WR031966

**Published:** 2022-12-27

**Authors:** Moritz Feigl, Stephan Thober, Robert Schweppe, Mathew Herrnegger, Luis Samaniego, Karsten Schulz

**Affiliations:** ^1^ Department of Water, Atmosphere and Environment Institute for Hydrology and Water Management University of Natural Resources and Life Sciences, Vienna Vienna Austria; ^2^ Department Computational Hydrosystems Helmholtz Centre for Environmental Research – UFZ Leipzig Germany

**Keywords:** regionalization, machine learning, rainfall‐runoff modeling, distributed models, transfer functions, optimization

## Abstract

Parameter estimation is one of the most challenging tasks in large‐scale distributed modeling, because of the high dimensionality of the parameter space. Relating model parameters to catchment/landscape characteristics reduces the number of parameters, enhances physical realism, and allows the transfer of hydrological model parameters in time and space. This study presents the first large‐scale application of automatic parameter transfer function (TF) estimation for a complex hydrological model. The Function Space Optimization (FSO) method can automatically estimate TF structures and coefficients for distributed models. We apply FSO to the mesoscale Hydrologic Model (mHM, mhm-ufz.org), which is the only available distributed model that includes a priori defined TFs for all its parameters. FSO is used to estimate new TFs for the parameters “saturated hydraulic conductivity” and “field capacity,” which both influence a range of hydrological processes. The setup of mHM from a previous study serves as a benchmark. The estimated TFs resulted in predictions in 222 validation basins with a median NSE of 0.68, showing that even with 5 years of calibration data, high performance in ungauged basins can be achieved. The performance is similar to the benchmark results, showing that the automatic TFs can achieve comparable results to TFs that were developed over years using expert knowledge. In summary, the findings present a step toward automatic TF estimation of model parameters for distributed models.

## Key Points

- This study shows the performance of automatic transfer function (TF) estimation with the Function Space Optimization (FSO) method
- We show that FSO is able to estimate TFs that perform similar to the mesoscale Hydrologic Model TFs in an ungauged setting
- This study represents a step toward automatic TF estimation for physically interpretable model parameters

## 1 Introduction

Large‐domain, spatially contiguous hydrological and land surface models are important tools for managing our water supplies. Hydrological information on the continental or global scale is needed to handle new emerging international and global water management challenges, which include topics like water allocation in international, national, and large river basins, operational flood forecasting services, global water security or the influence of climate extremes on water resources (Archfield et al., 2015). These applications are particularly challenging in areas without hydrologic measurements, which includes a majority of basins worldwide that are effectively ungauged (Hrachowitz et al., 2013). This results in the need for further development in large‐domain hydrological modeling to simulate water fluxes and states in both gauged and ungauged basins in different climates in a spatially consistent manner (Rakovec et al., 2019).

In 1982, Jim Dooge stated that “the parameterization of hydrologic processes to the grid‐scale of general circulation models is a problem that has not been tackled, let alone solved” (Dooge, 1982) and shortly after that Leavesley et al. (1983) concluded that optimization of distributed parameters of hydrological models is an “ill‐posed” problem due to the large number of degrees of freedom. Since then, model parameterization is still one of the major unsolved problems in hydrology (Blöschl et al., 2019). One way to potentially solve this problem is to relate hydrological model parameters/structures to landscape properties (e.g., K. J. Beven & Franks, 1999; K. Beven, 2002; Clark et al., 2016; Hundecha & Bárdossy, 2004; Samaniego et al., 2010). This approach is strongly related to the idea of regionalization—the geographical migration of hydrological model structures (Buytaert & Beven, 2009). This task is however nontrivial and Clark et al. (2017) still described it as one of the unsolved challenges for hydrological model parameter estimation.

One potential solution to this problem are parameter transfer functions (TFs) as mathematical expressions to formulate the relationship between model parameters and physiographic characteristics (e.g., elevation, slope, soil texture, vegetation characteristics, etc.) of the catchment (Hundecha & Bárdossy, 2004; Kumar et al., 2013; Samaniego et al., 2010). By defining TFs for all parameters, we expect to induce three attractive properties into the hydrological model:

1. The model has a significantly lower number of free parameters that is independent of the size of the model domain, facilitating parameter optimization.
2. The model can be transferred across time and space.
3. The model parameters reflect physical properties of the catchment and result in physical meaningful model states.

The first property would lead to a tremendous decrease in effort to set up and run distributed hydrological models. The problem of estimation of distributed parameters—an ill‐posed problem because of the large number of model parameters—would potentially be solved. The second property would allow for prediction in ungauged basins (PUB) and other time periods (Hrachowitz et al., 2013). Finally, the third property would allow the usage of model states and parameter fields to gain further insights into the hydrological properties and status of a catchment, which are generally extremely difficult or impossible to measure over such large areas (e.g., catchment‐wide soil moisture, soil properties, and evapotranspiration). The first two properties would result from TFs that are only dependent on a few numerical coefficients and predict discharge equally well in calibration and validation basins and time periods. The third property can potentially be achieved by the constrained setting of using TFs for parameters with a clear physical meaning and by relevant physiographic catchment characteristics as inputs.

Generally, the implementation of TFs for the estimation of distributed model parameters can be seen as a promising step toward adequately addressing critical water cycle science questions and global applications of hyperresolution hydrological and land surface models (K. J. Beven & Cloke, 2012; Wood et al., 2011). A corresponding requirement for hyperresolution models was also stated by Bierkens (2015): hydrological models should be able to make predictions “everywhere,” but the predictions should be “locally relevant.” For these reasons, Bierkens (2015) suggested that the multiscale parameter regionalization technique (MPR), which uses TFs at its input data's native spatial resolution to scale model parameters to the required spatial scale, could be a way forward. Overall, this will be an important step in the direction toward the application and parameterization of global hyperresolution models.

In a previous study, we developed a method to automatically estimate TFs from data called Function Space Optimization (FSO) (Feigl et al., 2020), which further developed ideas first proposed by Klotz et al. (2017) and Klotz (2020). FSO is based on a text‐generating neural network that is used to transfer the search for a best fitting TF in a continuous optimization problem. While other approaches consist of applying or adapting TFs by modifying their parameter (i.e., numeric coefficients) (e.g., Imhoff et al., 2020; Kumar et al., 2013; Pinnington et al., 2021; Samaniego et al., 2010), FSO can additionally change the functional form of the TF. So far, FSO was thoroughly tested on synthetic data by Feigl et al. (2020) and some initial results of a real‐world application were presented in Feigl et al. (2021).

The mesoscale Hydrological Model (mHM) (Kumar et al., 2013; Samaniego et al., 2010; Thober et al., 2019) is a distributed hydrological model that was already applied in numerous studies and for a wide range of different tasks, covering different hydroclimatic conditions (e.g., Imhoff et al., 2020; Jing et al., 2020; Kumar et al., 2013; Peichl et al., 2018; Saha et al., 2021; Samaniego et al., 2019; Thober et al., 2018). mHM is unique, as it has already TFs defined for all its parameters, which were chosen by Samaniego et al. (2010), Kumar et al. (2013), and Thober et al. (2019) based on pedo‐TFs from literature, a “step‐wise” method (Samaniego & Bárdossy, 2005) and a “trial‐and‐error” approach. This makes mHM an ideal model for testing FSO because we can compare the automatically estimated TFs with those chosen by expert knowledge and tested rigorously in multiple studies.

Besides the choice of model, choosing a benchmark study that applied mHM over multiple basins and in a PUB setting is important for objectively assessing the FSO performance. For this purpose, we chose the study by Zink et al. (2017) because it included a state‐of‐the‐art optimization and application of mHM over a large number of basins. Zink et al. (2017) estimated 100 global mHM parameters sets, that is, the numerical coefficients of all mHM TFs, for 7 large basins located in Germany with 5 years of data, which they then applied on 222 validation basins in Germany with a mean of 42 years of data.

This study assesses the performance and further develops the FSO approach and thus presents the next step in direction of regularizing the parameter space by using landscape information for distributed hydrological models. Its originality includes (a) further improvements of the FSO method, (b) a large‐scale application of automatic TF estimation using real‐world data and benchmark for comparison, and (c) a detailed description of challenges, potential limitations, and a way forward for automatic TF estimation.

## 2 Function Space Optimization (FSO)

### 2.1 Methodology

**Figure 1:**
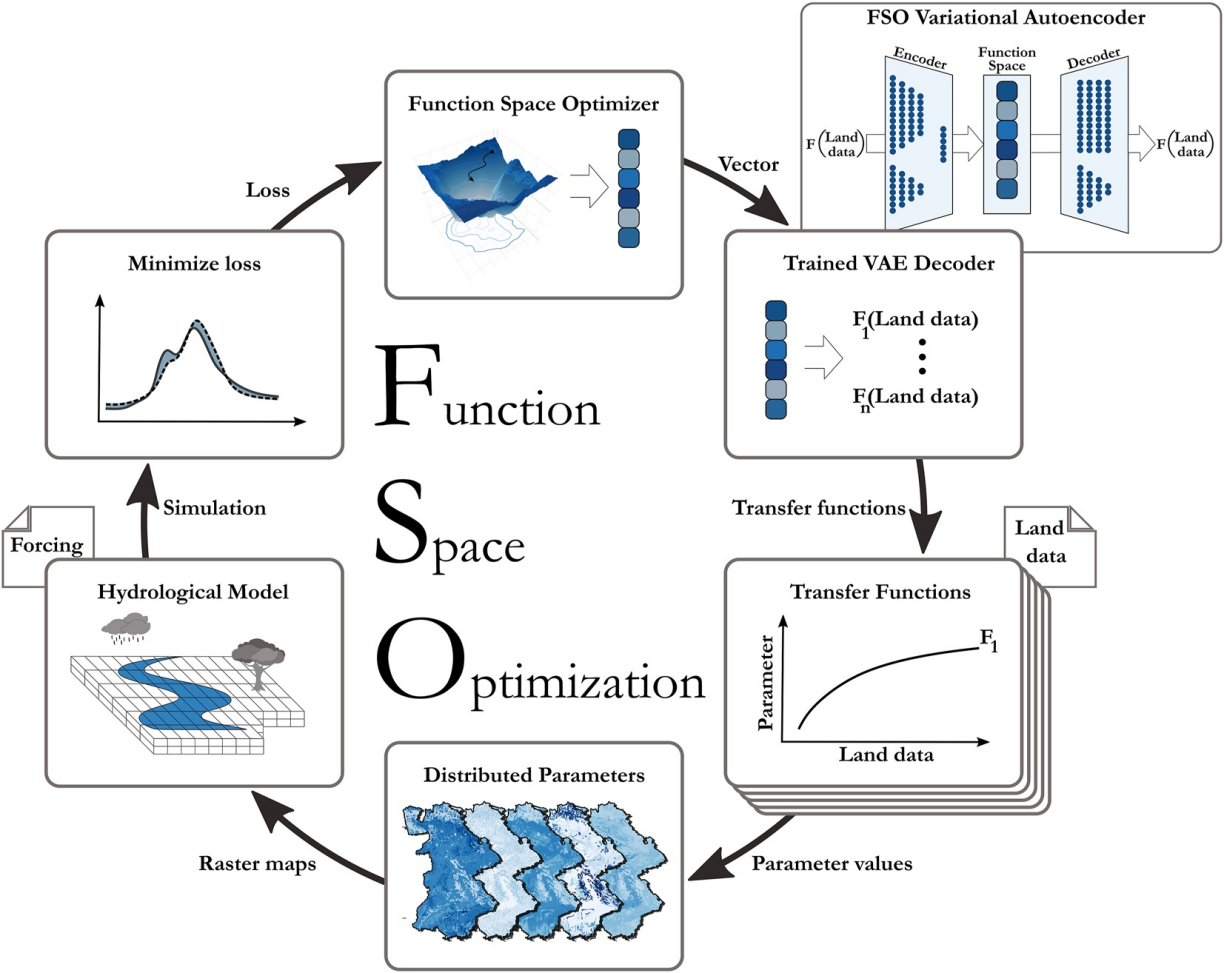
A depiction of the Function Space Optimization (FSO) loop. Starting from the top going clockwise: The optimizer selects the next point in Function Space that should be evaluated. The Function Space is derived by training the FSO variational autoencoder (VAE) using Transfer Functions (TFs) generated from a context free grammar that were evaluated using the land data of the study area. The trained VAE decoder generates the TFs that are associated with the chosen point in Function Space. The domain of the TFs comprises land data (e.g., elevation, slope, soil texture, and vegetation characteristics) and is used to generate parameter maps. These parameter maps are used as input for the distributed hydrological model to produce predictions (e.g., discharge and evapotranspiration). These predictions are then compared against observations using a loss function. Within this study, a mean loss is calculated over all observation gauges. This average loss is then used by the optimizer to decide on the next point in Function Space to be evaluated.

FSO is an optimization method for TFs of distributed models introduced by Feigl et al. (2020). It is based on the idea of transferring the search for a mathematical equation into a continuous optimization problem. This is achieved by training a neural network to generate TFs from a numeric vector whose values form the search space for an optimizer. The fully trained neural network provides a link between this search space and the corresponding TFs, so that we can apply one continuous optimization loop to find a best fitting set of TFs. All steps of the FSO optimization loop are shown in Figure 1. As in any continuous optimization problem, an optimization algorithm (optimizer, Figure 1 top) is used to find the point in a continuous vector space that minimizes or maximizes an objective function. Thus, the optimization algorithm is used to find the point in Function Space that produces TFs that lead to the best hydrological model fit.

The FSO workflow consists of two main parts, first, the neural network training, and second, the application of the optimization loop. The neural network used in the first part of the FSO workflow has a variational autoencoder (VAE; Kingma & Welling, 2013) architecture, consisting of an encoder network that transforms the inputs into a normally distributed numeric vector, and a decoder network that reconstructs the inputs from this vector representation (see Figure 1). We call this vector representation Function Space and the trained decoder can be used to generate TFs from any point of this vector space. The VAE network is trained to encode and decode the information of TF strings and their resulting parameter distribution given as quantiles. Data for training is generated using a context free grammar (CFG) (Knuth, 1965), which defines the possible structures, operators, functions, and variables that compose a TF. The variables are distributed (e.g., gridded) physiographic properties, that are the basis for parameter estimation. After generating TFs from the grammar, the quantiles of the resulting parameter values of each TF need to be estimated using data of the study area.

In the second part of the FSO workflow, the optimization loop (see Figure 1) is applied to find the point in Function Space that produces the best fitting set of TFs. In this loop, the optimizer is used to choose the next point in Function Space to be evaluated, which is consequently used by the trained VAE decoder to generate new TFs. Evaluating these TFs results in parameter maps that are utilized by the hydrological model to predict the hydrological variable(s) of interest. These predictions can then be used to compute a loss, which is based on a user‐defined objective function that is evaluated in each calibration basin, for example, NSE (Nash & Sutcliffe, 1970), KGE (Gupta et al., 2009). This loss is then used by the optimizer to choose the next point in Function Space for evaluation. Overall, the run time and computation cost of the FSO optimization loop depends on the hydrological model, because the computational resources of the other parts within the optimization loop are negligible. Therefore, the computational costs of one FSO iteration is similar to one model run for the whole study area.

To enable the unbiased estimation of universally applicable TFs, FSO uses two types of scaling. First, all physiographic properties are scaled to [0,1] before being applied in a TF. Second, the resulting TF values are scaled to the parameter bounds. Both scaling operations use min‐max scaling, which needs a minimum and maximum value for both the initial value range and the projected value range. The initial value range for the physiographic variables is chosen to be their physical bounds, for example, sand content [0, 100]. The initial value range for the TF values is chosen using the VAE training data and is the same for all generated TFs. A detailed description of FSO and all its preprocessing steps are given in Feigl et al. (2020).

We further developed the FSO VAE architecture to improve the encoding of long function strings. This should result in a Function Space with a smoother loss surface and thus making it a more adequate search space for optimization. A detailed depiction and description of the new network architecture is shown in Appendix A.

### 2.2 Linking FSO With the Mesoscale Hydrologic Model (mHM)

The mesoscale Hydrologic Model (mHM; Kumar et al., 2013; Samaniego et al., 2019, 2010; Thober et al., 2019) is a distributed hydrological model that simulates hydrological processes on a multi‐layer grid. It uses the MPR method (Samaniego et al., 2010; Schweppe et al., 2021) and thus uses TFs for all its parameters. The applied numerical approximations and conceptualizations are based on the HBV model (Bergström, 1995) and include the processes interception, snow accumulation, snowmelt, infiltration, surface runoff, soil water retention, runoff generation, evaporation, percolation, baseflow, and routing. It is also possible to calibrate mHM by optimizing the numerical coefficients of the mHM TFs, which is a far less complex optimization problem than calibrating the individual numerical parameter values. A detailed description can be found in Samaniego et al. (2010).

In this study we apply FSO to estimate new TFs for the mHM parameters *K*_*S*_ (saturated hydraulic conductivity [cm/day]) and *FieldCap* (field capacity [−]). These parameters affect the storage and conductivity of soil water and have a high sensitivity for streamflow estimation (Cuntz et al., 2015; Höllering et al., 2018). We want to minimize the effect of parameter dependency because this is the first large‐scale application of FSO with real‐world data. Therefore, we focus only on the estimation of these two TFs, which allows for a more in‐depth analysis of the results. The current version of mHM estimates *K*_*S*_ using a TF that was developed by Cosby et al. (1984):

(1) $$K_{S.mHM}=\gamma_{1}\exp\left(\gamma_{2}+\gamma_{3}\nu_{1}-\gamma_{4}\nu_{2}\right)\log\left(10\right),$$

which is a function of the sand *ν*_1_ and the clay *ν*_2_ content of the soil and numerical coefficients *γ*_1_to*γ*_4_. The current mHM parameter *FieldCap* is estimated using a TF by Twarakavi et al. (2009):

(2) $$FieldCap_{\mathrm{mHM}}=ThetaS\exp\left(\right.\gamma_{5}\left(\gamma_{6}+log10\left(K_{S}\right)\right)\log\left(vGenu_{n}\right),$$

where *ThetaS* is the saturated soil water content, *γ*_5_, *γ*_6_ are numerical coefficients and *vGenu*_*n*_ refers to the van Genuchten *n* model parameter (van Genuchten, 1980). While mHM *K*_*S*_ is only a function of observed physiographic properties, mHM *FieldCap* is also dependent on the other mHM parameters *ThetaS* and *vGenu*_*n*_.

## 3 Experimental Design

### 3.1 Benchmark and Study Data

The results of this study are compared to the results of Zink et al. (2017). In their study, they calibrated global mHM parameters, that is, the numerical coefficients of all mHM TFs, using 5 years of data for 7 large basins in Germany. The calibration was conducted using the dynamically dimensioned search (DDS) optimization algorithm (Tolson & Shoemaker, 2007) with 2,000 iterations and was performed 100 times in each of the 7 basins. From these, they selected 100 parameter sets that had a Nash‐Sutcliffe model efficiency (Nash & Sutcliffe, 1970) exceeding 0.65 in all 7 basins. Finally, they validated these 100 parameter sets using 42 years of data from 222 smaller basins across Germany. This provides an estimate of the mHM performance in an ungauged setting. We choose the same data and experimental setup as Zink et al. (2017) to make results comparable.

**Figure 2:**
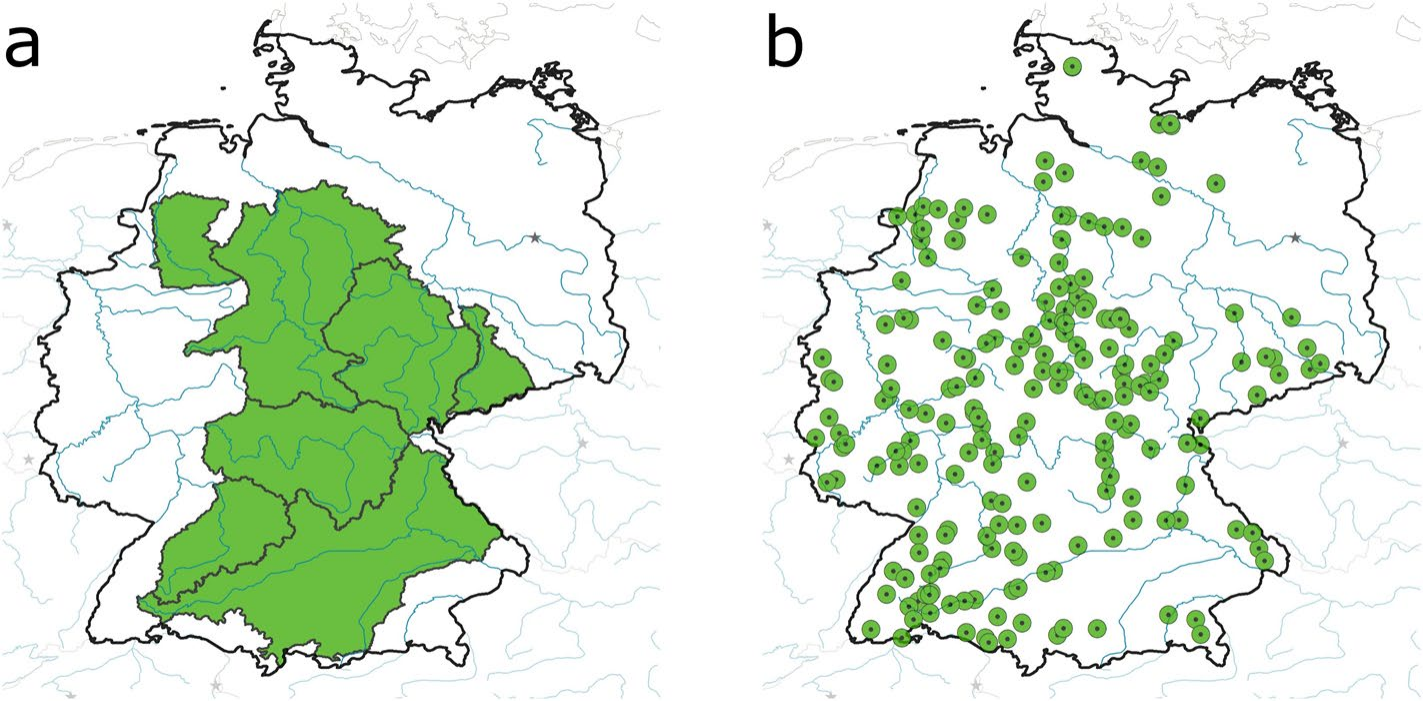
Overview of the study basins in Germany. (a) The seven large basins used for calibration. (b) The 222 validation basins shown with the position of their gauges.

All meteorological forcings, physiographic properties, and discharge observations are taken from Zink et al. (2017), which also includes a detailed description of the preceding data preparation. The study basins consist of 7 large basins used for calibration and 222 smaller validation basins. The calibration basins have a size range of 6,200 km^2^ to 47,500 km^2^, while the validation basins have a size range of 100 km^2^ to 8,500 km^2^. The area of all calibration basins is shown in Figure 2a and the outlets of all validation basins are shown in Figure 2b. Of these 222 validation basins, 80 are located outside and 142 are located inside the calibration basins area.

The available variables for TF estimation consist of six physiographic properties on a 100 × 100 m grid: sand content in percent (*ν*_1_), clay content in percent (*ν*_2_), mineral bulk density in g/cm^3^ (*ν*_3_), aspect in degree (*ν*_4_), slope in degree (*ν*_5_) and elevation in m (*ν*_6_). The variables *ν*_4_, *ν*_5_, and *ν*_6_ were derived from a 50 m digital elevation model acquired from the German Federal Agency for Cartography. The soil properties are based on a digitalized soil map of the German Federal Institute for Geosciences and Natural Resources and contain information for different soil horizons.

The meteorological forcings that are used for running mHM consist of daily fields of precipitation and maximum, minimum, and average temperature, which were derived from local observations from the German Weather Service (Deutscher Wetterdienst, DWD). Daily streamflow data was provided by the European Water Archive and the Global Runoff Data Center. Land cover information was taken from the CORINE land cover scenes of the years 1990, 2000, and 2006.

### 3.2 mHM Setup and Objective Function

Following Zink et al. (2017), the resolution of the mHM model is 4 × 4 km and each simulation is conducted with a 5‐year spin‐up period. Calibration data consists of the years 2000–2004 of the 7 large basins. The performance of these 7 large basins is validated in the time period 1965–1999. Transfer in space and time is tested by using data between 1955 and 2009 of 222 validation basins, resulting in a mean simulation time period of 42 years and a minimum simulation period of 10 years. In summary, one FSO optimization loop iteration consists of running mHM in the 7 training basins for 10 years (5 years spin‐up + 5 years training), which is done in parallel and thus as fast as one mHM run. After reaching a minimum value of the objective function in the FSO optimization loop, mHM using the FSO estimated TFs is applied to the 7 training basins in validation time and the 222 validation basins.

The objective function Φ for evaluating the mHM performance is chosen to be a combination of the NSE and log NSE criteria in form of a power mean:

(3) $$\Phi ={\left(\frac{1}{2}\sum_{i=1}^{2}\phi_{i}^{p}\right)}^{\frac{1}{p}},$$

with *ϕ*_1_ = *NSE*(*Q*_*obs*_, *Q*_*sim*_), *ϕ*_2_ = log *NSE*(*Q*_*obs*_, *Q*_*sim*_), *Q*_*obs*_ and *Q*_*sim*_ the observed and simulated discharge, and *p* the exponent of the *L*_*p*_ norm (Duckstein & Opricovic, 1980) that was chosen to be *p* = 6. This objective function was chosen by Zink et al. (2017) as it ensures equal improvement of both criteria during a multi‐objective optimization.

### 3.3 FSO Setup

FSO is applied to identify the two TFs and their parameters to regionalize *K*_*S*_ and *FieldCap* and simultaneously to optimize the numeric coefficients of all other mHM TFs. The resulting numeric vector, which represents all optimizeable values, consists of 12 dimensions for the two TFs (two 6‐dimensional Function Spaces) and 59 dimensions for the remaining global TF parameters of mHM.

Before applying the FSO optimization loop, it is necessary to train the FSO VAE using a set of function strings and their resulting parameter distribution. This training data was generated using a CFG that included the physiographic variables, numeric values, the operators +, −, *,/, and a range of mathematical functions: the exponential function, the logarithm function with base *e* or 10, trigonometric functions (sin, cos, and tan), and their arcus and hyperbolic versions, the square root and power functions. The numeric values were chosen to be in the range [−3, 3], discretized with a 0.05 step size. This interval was chosen to allow for a wide range of parameter values to be generated while keeping the search space size manageable. Since we use physiographic variables that are scaled to the range [0, 1], the numeric values thus can be up to three times larger. The CFG is shown in Appendix B, which includes all its building blocks and structural components. This CFG is used to generate 45 million unique TFs that are used to train the FSO VAE. To make our results comparable to Zink et al. (2017), we trained two different FSO VAEs, one for each regionalized parameter. The difference between those two are the variables that are used to generate functions: while *K*_*S*_ only uses observed physiographic properties, *FieldCap* can also use the mHM parameters *K*_*S*_, ThetaS (*ν*_7_) and *vGenu*_*n*_ (*ν*_8_) as potential inputs. We estimated the distribution for each TF by applying it to approximately 100,000 grid points sampled from our training basins. Hence, for each TF we computed 100,000 parameter values that we used as basis to estimate the TF quantiles that represent the parameter distribution resulting from the TF. These quantiles together with their corresponding TF strings are the in‐ and outputs of the FSO VAE. Further technical details of the FSO VAE, including all bounds used for scaling and the training details, are given in Appendix B.

FSO aims to find the set of TFs that results in the best performance in all available basins. Therefore, Feigl et al. (2020) defined the FSO loss function *f*_*loss*_ for *I* basins to be a weighted mean with more weight on basins with bad performance:

(4) $$f_{\mathrm{loss}}=-\frac{\sum_{i=1}^{I}w_{i}\Phi_{i}}{\sum_{i=1}^{I}w_{i}}+\lambda \left(TFs\right),\;\text{with}\;\text{each}\;w_{i}=\sup\;\Phi -\Phi_{i}$$

Here, Φ_*i*_ is the value of the objective function for basin *i*, *w*_*i*_ is the corresponding weight, and *λ*(*TFs*) is the penalty for TF lengths. The weights are computed by using the supremum of Φ, which is 1 for the above described objective function that is based on NSE and log NSE. The penalty function *λ* is a function of the number of variables, numerics, operators, parenthesis and functions used in the TFs (i.e., number of tokens) and can be computed with $\lambda \left(TFs\right)=\frac{1}{M}\sum_{m=1}^{M}\alpha \;length\left(TF_{m}\right)$, where *M* is the number of estimated TFs and *α* was chosen to be 0.001 (Feigl et al., 2020). This value was chosen as it results in *λ*(*TFs*) values around 0.01–0.1 (assuming TF lengths between 10 and 100 tokens), which is a reasonable penalty for an NSE type objective function with values ≤1. Overall, this loss function results in a weighted average of objective function results that are always in the range of the objective function, which gets modified by the penalty function to penalize overly complex TFs.

In our preliminary test runs, we used the DDS algorithm (Tolson & Shoemaker, 2007) for optimizing in Function Space. However, we noticed that it converged extremely fast (<500 iterations) potentially as a result of the algorithm's abidance in local minima. Thus we decided on mainly using the shuffled complex evolution (SCE) algorithm (Duan et al., 1992) for this study. For both optimizers, the optimization is applied for a minimum of 2,000 iterations and a maximum of 5,000 iterations. It will be stopped if after the minimum required iterations there is no further improvement for 1,000 iterations. After one of the stopping criteria was reached, all numeric coefficients that are present in the two TFs are further optimized with 100 iterations of the Genetic Algorithm (Holland, 1975, GA). This additional optimization allows only a ±5% change of the numeric coefficients found by the VAE‐based SCE/DDS optimization and represents an adjustment that is not bound by the discretization of the numeric coefficients in the FSO VAE. We chose the GA algorithm since it was easy to implement. For this polishing step the choice of algorithm is not crucial, as the optimization problem is only dealing with a handful of numeric values with a narrow range of possible values.

### 3.4 Optimization Budget and Evaluation Criteria

The FSO method is applied 5 times to the 7 calibration basins, resulting in five independent optimization runs. Four optimization runs will use the SCE algorithm (run 1–4), while one will use the DDS algorithm (run 5). Their performance will be evaluated using the *f*_*loss*_, NSE, log NSE, and KGE values.

The validation results of Zink et al. (2017) will be compared to the best performing FSO optimization run. The decision of the best performing optimization run will be based on the NSE, log NSE, KGE and percentage bias (PBIAS; Sorooshian et al., 1993) values in 20 randomly sampled validation basins (approximately 10% of the validation basins). The definition of all performance metrics is given in Appendix C. The random validation sample, which is used to define the best FSO run, will be drawn from a stratified Budkyo curve (Budyko, 1974) to adequately represent the range of different climates in the study area. Since the results of Zink et al. (2017) are the NSE distributions for all validation basins resulting from the 100 parameter sets, the minimum, maximum, 5% quantile, 95% quantile, and median NSE of each basin will be used for comparison.

The sampled validation approach was chosen to test whether it is possible to find the best model run given a very small subset of basins and only compare this best run with the results of Zink et al. (2017). This approach simulates a real‐world FSO application that would include only a small set of validation basins and then an application in a large set of ungauged basins. If the results of the sampled validation and the full validation are very similar it would point to robustness of the FSO performance. Similarly, if the results would differ it would point to possible issues in transferability of FSO estimated TFs.”

Different TFs can potentially result in similar parameter values, thus we will also compare the FSO parameter fields to the mHM default parameter fields of the two parameters *K*_*S*_ and *FieldCap*. For this purpose, the 7 calibration basins will be used, as they present a contiguous field covering a large part of Germany.

## 4 Results

### 4.1 Comparison of Optimization Runs

**Figure 3:**
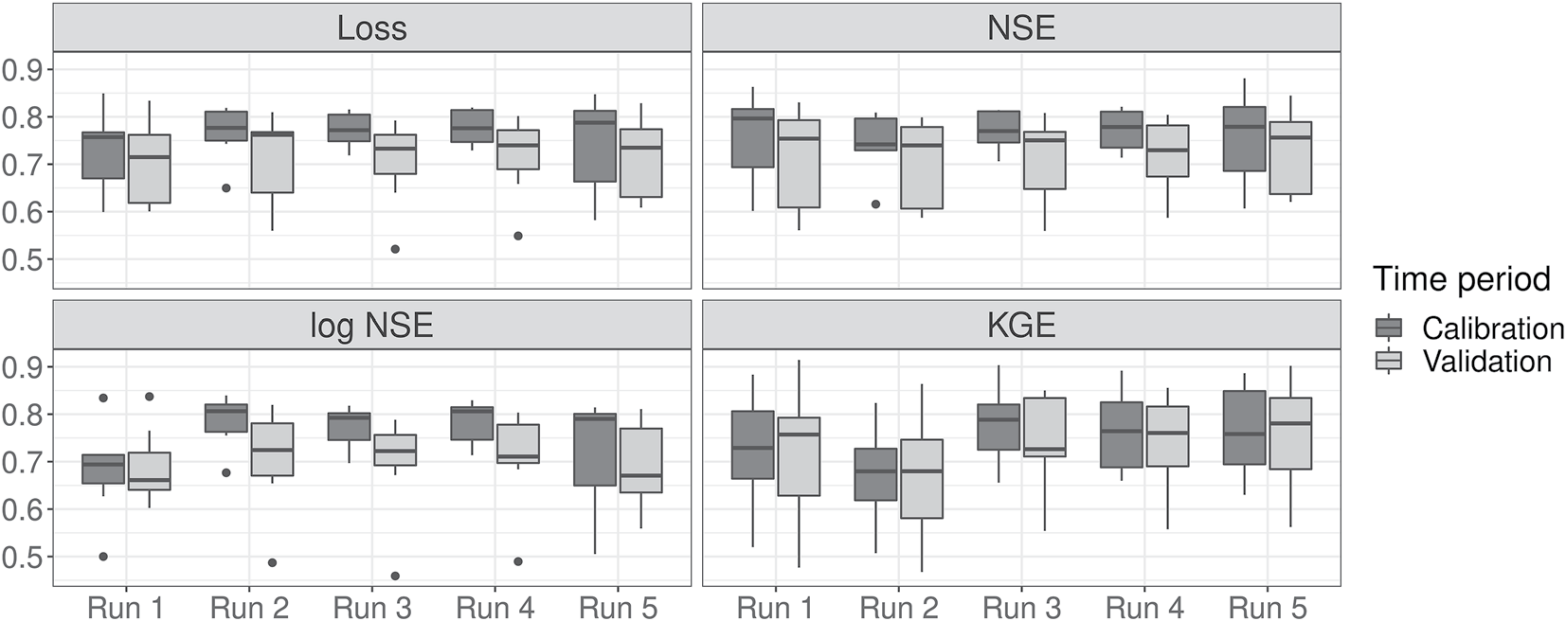
Boxplots of different performance metrics of the 5 Function Space Optimization runs in the 7 training basins for the calibration (2000–2004) and validation (1965–1999) time periods.

This section presents the results of the final parameter sets of each FSO optimization run. The progression during optimization of all 5 FSO runs is shown in Appendix D. These 5 FSO runs resulted in a total of 19,867 performed mHM simulations, where one simulation equals an mHM run for the 7 training basins. Figure 3 shows boxplots with the performance of the final parameter sets of all FSO runs for the calibration time period (2000–2004) and validation time period (1965–1999) in the 7 calibration basins. This also includes the KGE values, which were not part of the loss function and thus had no influence on the optimization. Run 2–4 show very similar calibration performance, which differs from run 1 and 5 performances. The median calibration NSE values of run 1 with 0.76 and run 5 with 0.79 are similar to the run 2–4 medians with a mean of 0.76, but have a much larger variance (*σ*_*run*1*&*5_ = 0.1, *σ*_*run*2*−*4_ = 0.03). This is also the case for run 5 calibration log NSE values, whereas run 1 calibration log NSE values also have a lower median compared to all other runs. Log NSE values are especially similar in runs 2–4 with median values ranging between 0.79 and 0.81. The KGE values show slightly different behavior. Run 2 KGE values for the calibration period are lower with a median of 0.68 compared to run 3 and 4 a mean median KGE of 0.78.

The difference between calibration and validation time period of KGE values is negligible with a median difference of −0.01. The differences of NSE values with a median of −0.03 and the differences of log NSE values with a median of −0.04 are more pronounced.

Table 1 shows the FSO estimated TFs for all runs. To ease readability, they do not include the scaling factors for variables and TFs which are applied to compute the parameter values. Hence, it is difficult to estimate the value range that results from these TFs solely from the given function, but it shows the complexity, non‐linearity, and used physiographic properties.

**Table 1:** Function Space Optimization Estimated Transfer Functions for the Mesoscale Hydrologic Model Parameter *K*_*S*_ and *FieldCap* for the Optimization Runs 1–5 and Their Corresponding Value Ranges

Run	*K*_*S*_ (cm/day)	Value range	*FieldCap* (−)	Value range
1	*ν*_6_ − 3.009(*ν*_3_+cosh(*ν*_5_))	[1.1, 243.7]	$\frac{\cos\left(\nu_{2}\right)}{\nu_{3}}+\tanh\left(\nu_{5}\right)$	[0.250, 0.299]
2	*ν*_4_ − 3.223*ν*_3_ − 2.72	[1.1, 67.6]	$\cos{\left(\cosh{\left(\nu_{6}\right)}^{2}\right)}^{2.816}$	[0.222, 0.228]
3	−3.182 − cosh(*ν*_3_)	[105.4, 117.1]	*K*_*S*_ − (2.682 + *ν*_7_ cosh(*ν*_3_))	[0.100, 0.139]
4	*log*10(*ν*_4_) − 3.167	[1.1, 177]	*ν*_4_ − *ν*_4_ − 3.286	[0.100, 0.100]
5	$\frac{0.101}{\left.\left(\nu_{2}\arcsin\left(\nu_{1}\right)\;-\;1.0\right)\right)}-\left(\nu_{2}+\cos\left(\nu_{6}\right)\right)$	[16.2, 102.9]	*ν*_5_	[0.222, 0.311]

*Note.* Some functions were simplified to ease readability and thus do not necessarily reflect the direct VAE output. All physiographic properties (*ν*_1−6_) and mHM parameters (*K*_*S*_, *ν*_7_) are scaled to [0,1] before being used in these TFs. The values resulting from these TFs are scaled to the parameter value range to generate the final parameter sets for the model. The physiographic properties are sand content (*ν*_1_), clay content (*ν*_2_), bulk density (*ν*_3_), aspect (*ν*_4_), slope (*ν*_5_), elevation (*ν*_6_), saturated hydraulic conductivity (*K*_*S*_) and ThetaS (*ν*_7_).

The estimated TFs show different lengths and levels of complexity. There is no specific physiographic characteristic, which is part of all TFs for either of these two parameters. Even in the TFs of run 2–4, which are very similar in performance, no variable was used in every run. The FSO VAE is constrained to not produce constant functions. Interestingly, this was bypassed by generating a function that includes *ν*_4_−*ν*_4_, which results in a constant value for the run 4 *FieldCap* TF.

### 4.2 Sampled Validation

**Figure 4:**
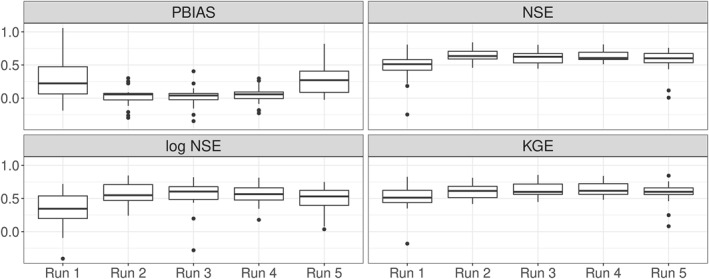
Boxplots of different performance metrics of the 5 Function Space Optimization runs in 20 randomly sampled validation basins. The sampled validation basins were randomly drawn from a stratified Budyko curve and thus represent the full range of climates in the study region.

The run used for the benchmarking test with Zink et al. (2017) is based on validation performance in 20 sampled basins shown in Figure 4. The previous results already showed that there is a distinct difference in performance in run 1 and 5 compared to run 2–4. This is again visible in the results of the sampled validation, especially in the PBIAS values. Run 1 and 5 have large positive median PBIAS values of 22.3% and 27.1%, indicating model overestimation bias. On the other hand, run 2–4 simulations have low median PBIAS values of 5.6%, 4.0%, and 5.7%, respectively.

Run 2 simulations result in the highest NSE values with a median of 0.63. Median log NSE values of runs 2–4 are again very similar with values of 0.55, 0.60, and 0.57, with the main difference being that run 2 is the only run that does not include outliers. Median KGE values of runs 2–4 are also very similar with values of 0.61, 0.60, and 0.62. With the highest NSE, no outliers in the log NSE, and equally high KGE as the other runs, run 2 was chosen as the best model run that will be compared to the benchmark in all 222 validation basins.

### 4.3 Validation and Benchmark Evaluation

**Figure 5:**
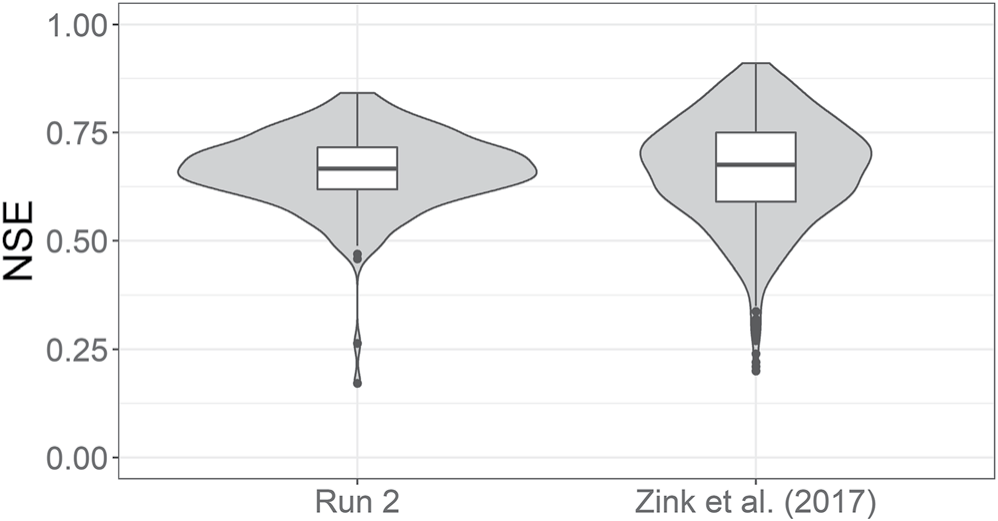
Violin plots and boxplots of NSE values of the 222 validation basins for Function Space Optimization run 2 and the Zink et al. (2017) values. The Zink et al. (2017) values consist of the resulting minimum, maximum, 5% quantile, 95% quantile and median NSE of their applied 100 parameter sets.

To assess the performance of FSO, the FSO run 2 NSE results are compared to the minimum, maximum, 5% quantile, 95% quantile, and median NSE of the 100 parameter sets applied by Zink et al. (2017) for the 222 validation Basins. Figure 5 shows the resulting violin plots and boxplots of NSE values. The NSE medians are almost equal for both experiments (run 2 = 0.67, Zink et al. (2017) = 0.68). The main difference can be seen in the NSE variance which is lower in the FSO run 2 results (*σ*_*run*2_ = 0.008, *σ*_*Zinketal*(2017)_ = 0.015) and the numbers of outliers of the Zink et al. (2017) simulations. Testing the differences between these two NSE value distributions using a Kruskal‐Wallis test (Kruskal & Wallis, 1952) did not show a significant difference (*p*−*value* = 0.457). While the run 2 results are the NSE values of one specific parameter field, the Zink et al. (2017) NSE values are the results of 100 different parameter sets. Thereby, they represent the overall behavior of the original mHM TFs, but individual points can not directly be compared to the FSO results.

**Figure 6:**
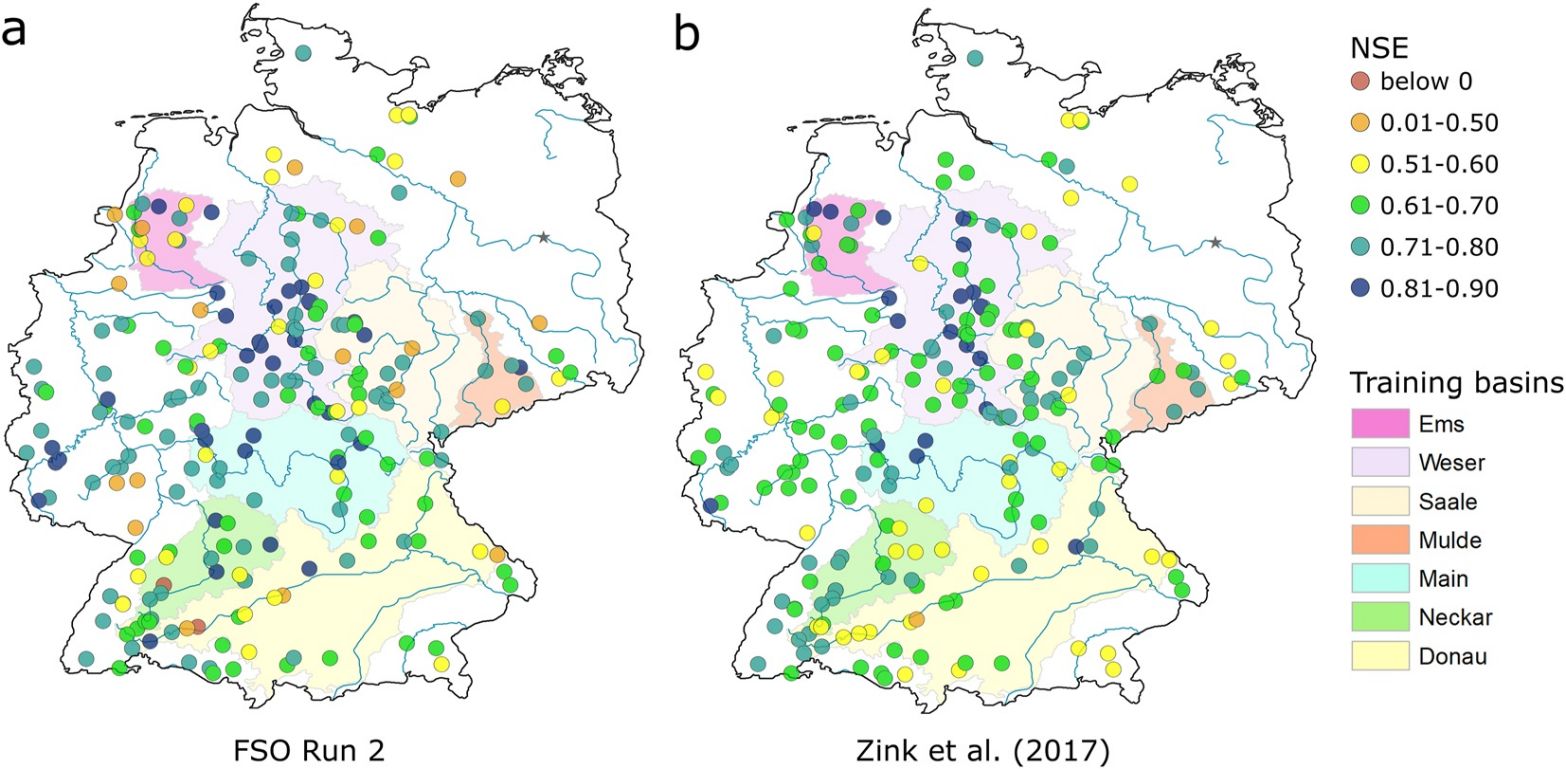
Maps of NSE for the 222 validation basins in Germany. (a) Function Space Optimization run 2 results. (b) Zink et al. (2017) median results.

Figure 6 shows the spatial NSE patterns of FSO run 2 and median Zink et al. (2017) results. The benchmark median NSE seems to be slightly higher in the northernmost basins, while FSO run 2 shows higher NSE values in western and central Germany. Other than that, no distinct spatial pattern can be observed. The median NSE of basins that lie inside the calibration basins is 0.68 and nearly equal to 0.66 for basins outside. The same is true for Zink et al. (2017) results with a median NSE inside the calibration basins of 0.68 and outside of 0.67.

To further assess the FSO performance, the relationship between resulting NSE values and the validation basins climate, basin area and mean altitude is analyzed. For this task, the climate is represented by the basins' Aridity index (PET/P). Testing for dependency using a linear regression shows a significant positive association of NSE with the basin area (coefficient = 2.155 × 10^−5^, *p*‐value <10^−7^) and a negative association of NSE with the mean altitude (coefficient = −1.2 × 10^−4^, *p*‐value = 0.006), but no existing association of NSE with aridity (*p*‐value = 0.113).

### 4.4 Comparison of Parameter Fields

**Figure 7:**
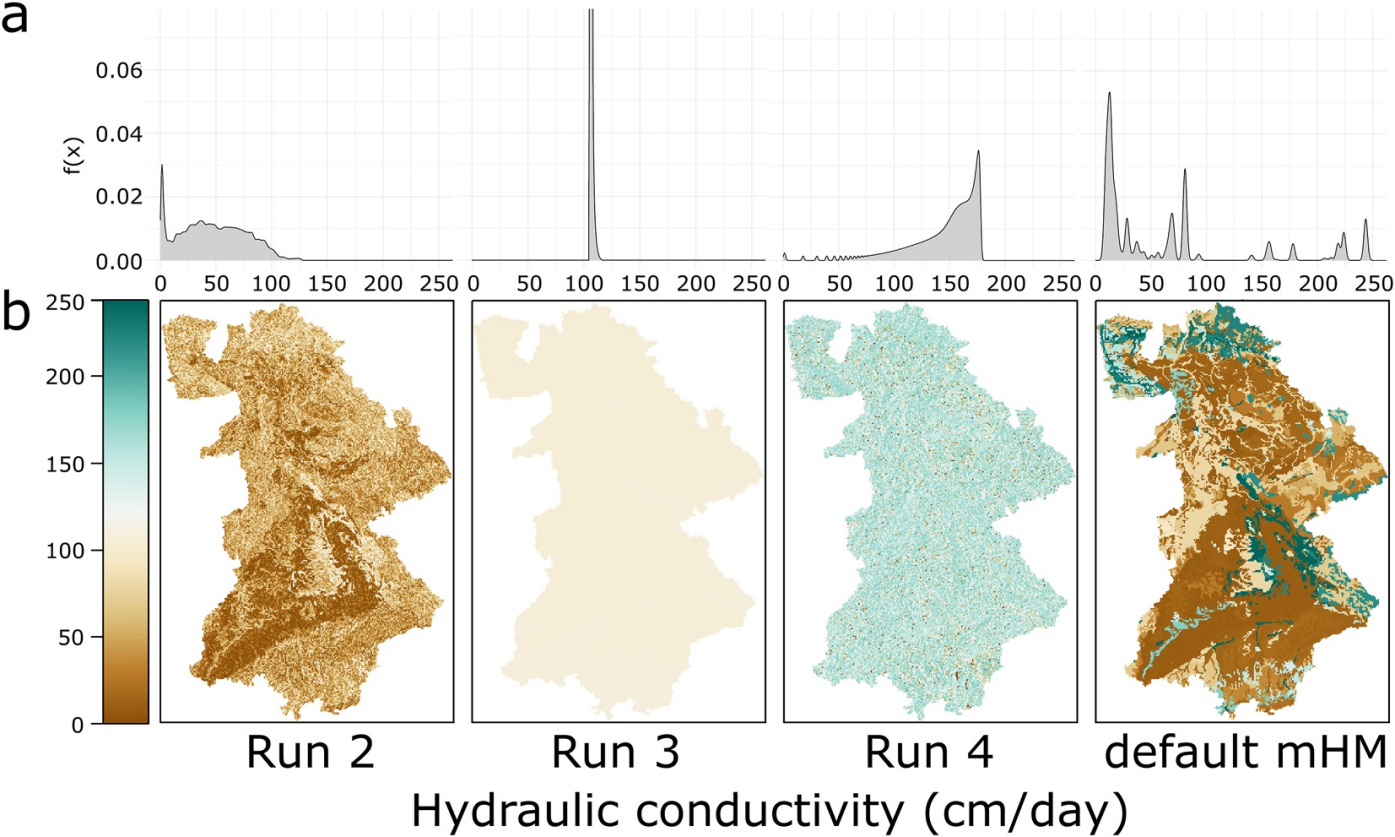
Resulting parameter values for the mesoscale Hydrologic Model (mHM) parameter *K*_*S*_ (saturated hydraulic conductivity, cm/day) for three Function Space Optimization (FSO) runs and the default mHM parameter set for the 7 calibration basins. (a) Parameter distributions of FSO run 2–4 and mHM default parameter and (b) parameter fields of the 7 calibration basins.

Finally, the FSO generated parameter fields are examined and compared to the default mHM parameter fields. In addition to the best performing run (run 2), we will examine the resulting parameter fields of run 3 and 4, which produced nearly equally good simulations. Figure 7a shows the *K*_*S*_ (cm/day) parameter distributions and Figure 7b the corresponding parameter fields. These parameter fields have very different characteristics. Only the spatial patterns of run 2 and the default mHM parameter is somewhat similar, however, run 2 does not have areas with high *K*_*S*_ values (>150 cm/day). The TF of run 3 produces nearly constant *K*_*S*_ values of around 110 cm/day, while the run 4 TF results in values mostly between 150 and 200 cm/day with isolated lower outliers distributed over the whole area.

**Figure 8:**
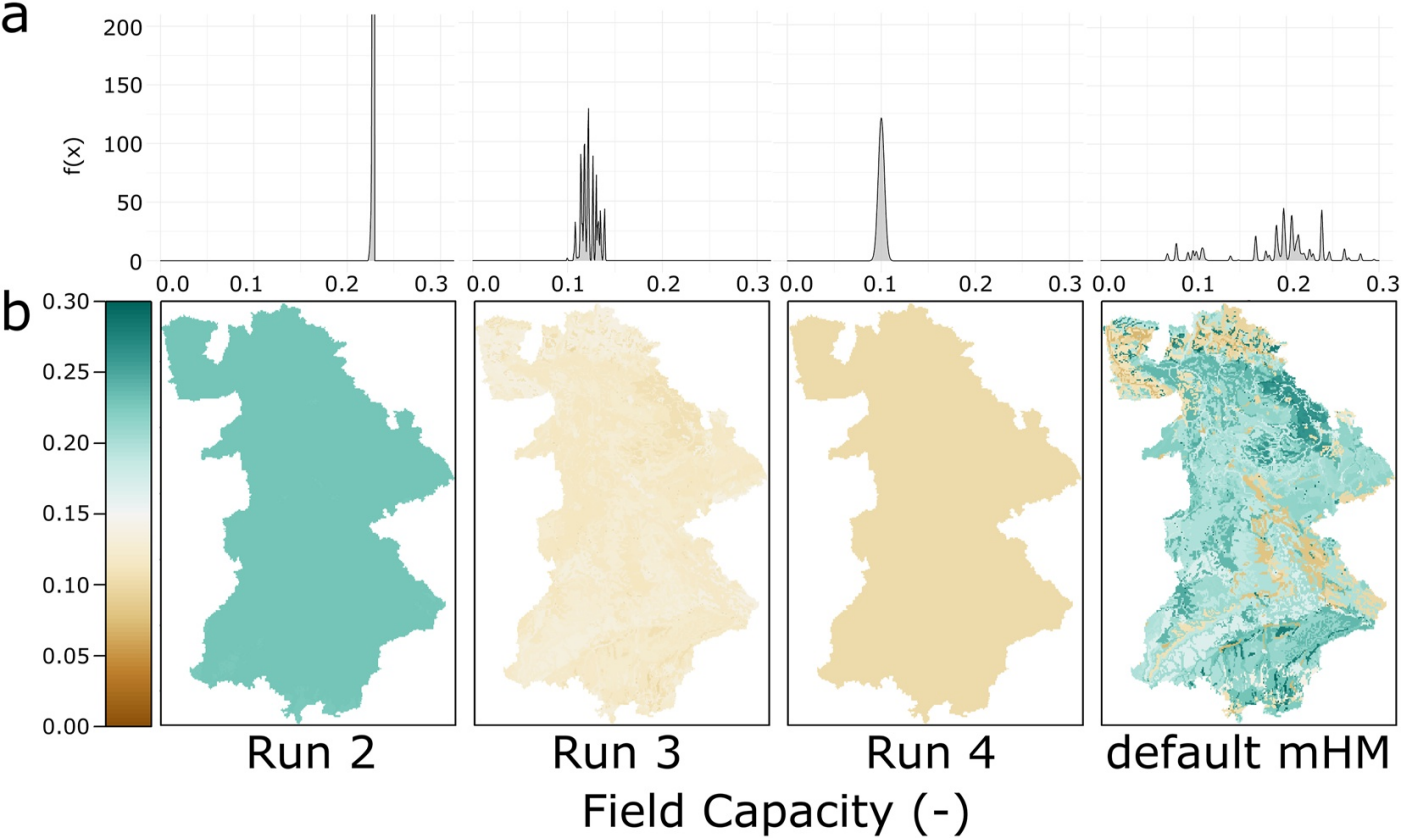
Resulting parameter values for the mesoscale Hydrologic Model (mHM) parameter *FieldCap* (field capacity, −) for three Function Space Optimization (FSO) runs and the default mHM parameter set for the 7 calibration basins. (a) Parameter distributions of FSO run 2–4 and mHM default and (b) parameter fields of the 7 calibration basins.

Figure 8a shows the *FieldCap* [−] parameter distributions and Figure 8b the corresponding parameter fields. The TFs of two of the FSO runs, run 2 and run 4, predict a constant value: *FieldCap*_*run*2_ = 0.222, *FieldCap*_*run*4_ = 0.100. Run 3 values show more variability with values in the range of [0.10, 0.14]. Default mHM values have a median of 0.20 are thus generally higher than run 3 and run 4 FSO values, but lower than run 2 values. The default mHM TF for *FieldCap* results in values with a much higher variance compared to all FSO estimated *FieldCap* TFs.

## 5 Discussion

Estimating distributed parameter fields for hydrological models with TFs is potentially able to significantly reduce the number of free model parameters, make the model transferable across time and space and produce physically meaningful parameters and model states. The presented results show that with the current FSO approach we are able to induce the first two properties in the model. Using the FSO estimated TFs greatly reduces the complexity of optimization compared to a model whose parameters are estimated for each pixel or each spatial modeling unit. Furthermore, the model had only a slight reduction in performance when applied in validation basins. This is interesting and notable as it represents a PUB problem that is inherently difficult for hydrological models to solve. Both these goals are reached by using only 5 years of data for training. However, these years include a year with high‐impact flood events in central Europe (2002) and a year with a significant drought event (2003). The findings regarding the third property will be discussed in a following paragraph.

Of the 5 FSO runs, 3 performed nearly equally well, while 2 runs had lower performance and already showed issues with convergence during optimization. One of these 5 runs used the DDS, which we already expected to have convergence issues. However, it should be noted that due to our constraints on computational resources this single DDS trial can not be seen as an algorithm comparison and thus the results cannot be interpreted in that regard. Generally, our results show that optimization in Function Space is difficult and could potentially still be improved by a VAE that is able to produce a smoother Function Space for optimization. The current version leads to similar performance results in three out of four times when using the SCE for optimization. Reducing the stopping criteria to 500 iterations without improvement would most likely result in discarding a run with convergence issues in an early stage of the optimization. Comparison of the convergence behavior of FSO to other studies on large‐scale parameter estimation is not possible, since they are neither reported for mHM (e.g., Dembélé et al., 2020; Rakovec et al., 2016) nor for other models (e.g., López López et al., 2017; Mizukami et al., 2017).

The FSO estimated TFs perform as well as the benchmark predictions by Zink et al. (2017) which is an astonishing result considering its implications. The TFs used by Zink et al. (2017) were developed over a span of 10 years using a large amount of literature and expert knowledge and were developed in some of the German basins that are part of this study (Kumar et al., 2013; Samaniego et al., 2010, 2019; Thober et al., 2019). Furthermore, the benchmark represents the results of the best 100 parameter sets after an overall of 1.4 million optimization iterations, which are around 70 times more iterations compared to the presented study. Additionally, the optimization problem solved by Zink et al. (2017) was much simpler as TF structures were fixed and only coefficients were optimized.Also their single‐site independent calibrations did not lead to complex multi‐modal objective functions. Hence, this benchmark is more like an upper bound that we aim to reach with an automatic setting, rather than a threshold we certainly have to cross. The current state of FSO is able to estimate TFs that reach this upper bound and would allow for estimating TFs for other models without the work that was necessary for developing the mHM TFs and by using considerably less computational resources. This will drastically reduce the time to implement TFs for a new model that is coupled with the FSO and MPR method.

Hydrological model performances are strongly dependent on the basins and the available data quality. Therefore, it is important to use a benchmark to adequately interpret the results of a study. For this reason, we chose the study of Zink et al. (2017), as it had the necessary scope and used the same model. The Zink et al. (2017) benchmark setup was the best possible available option but increased the difficulty of the task for two reasons. One reason was the fact that only quantiles of performance values were available from Zink et al. (2017). We do not know whether run 2 actually has a higher performance than each individual parameter set of Zink et al. (2017). The second reason can be found in the selection of calibration basins. Zink et al. (2017) used 7 very large basins for calibration because they only had to optimize a small set of numerical parameters of existing TFs. This is a much more constrained optimization problem compared to the estimation of structure and numerical coefficients of TFs. As each basin results in one loss value, the most pronounced feedback for optimization would be reached by using a set of basins that have a high variance of physiographic properties between them, but a low variance inside each basin. Hence, only a few extremely large basins with a high internal variance of physiographic properties are not the best starting point. In summary, a larger set of smaller or mixed‐size basins with a wide range of physiographic characteristics used for calibration would most likely be advantageous for estimating TFs. Interestingly, FSO‐mHM still performs as well as the median of 100 parameter sets with this non‐ideal selection of calibration basins, pointing to the fact that there is still potential for improvement from a different calibration setup.

From the results, it is evident that physical interpretation of the estimated TFs and the resulting parameter fields is still difficult at this point. This is indicated by the three FSO runs (run 2–4) having a very similar discharge simulation performance, while having very different parameter fields. However, these different TFs can provide further insights into the model structure and guide further model development. Looking at different parameter values estimated by the FSO TFs, it is interesting that all of them produce constant values or nearly constant values for the field capacity parameter. Looking into the model states, we could observe that soil water content was usually above field capacity in the FSO models. This is equivalent to a model simplification, showing that the study region, which does not include arid basins, can be represented without using the parameter *FieldCap*. These parameter sets will most likely perform poorly in arid regions. Similarly, the predicted saturated hydraulic conductivity values were diverse, with only FSO run 2 showing similar patterns compared to the default mHM parameters. *K*_*S*_, unlike *FieldCap*, is not directly used by mHM but is used to compute the model parameter *Kperco*—the *K* factor for percolation that controls the amount of water that flows between soil layers. This conceptualization of the parameter is on one hand influencing its physical interpretation, which potentially differs from the definition of the saturated hydraulic conductivity, but on the other hand, potentially makes it easier to find a suitable relationship to the physiographic properties of a catchment. This is especially important as saturated hydraulic conductivities at larger scales are difficult to measure or estimate with PedoTFs (Zhang & Schaap, 2019).

While we could have just chosen run 2 parameter fields for comparison, we included the slightly lower performing runs as well to show that parameter equifinality still exists when applying FSO. This may be primarily a result of using only large basins for calibration, which only give coarse feedback during optimization. In our opinion, this equifinality will most likely be strongly reduced if an appropriate set of calibration basins are selected. This was not possible in this study because we wanted to have a comparable benchmark. As mentioned above—ideally, these calibration basins should consist of a larger number of basins with a high variance of physiographic properties between them, but a low variance inside each basin. Still, further constraints using additional boundary conditions, for example, soil moisture or ET‐fluxes, are potentially helpful for predicting physically sound parameter values. Additionally, a wider range of physiographic properties used in FSO TFs would produce a larger search space and potentially better performing TFs. This could include inputs derived from existing ones, for example, the Topmodel index $\ln\left(\frac{a}{slope}\right)$ (K. J. Beven & Kirkby, 1979), inputs that have shown relevance for other hydrological or soil science prediction tasks, for example, the relevant inputs used in the SoilGrids regression trees (Poggio et al., 2021), or other gridded inputs that represent vegetation, soil and climatic properties of the catchments. Furthermore, for future studies, the number of TFs estimated with FSO should be increased because we could show that optimizing two TFs is feasible.

Recently, there have been two other studies that derived distributed model parameters from physiographic attributes which both applied the Variable Infiltration Capacity model (VIC, Liang et al., 1994) over the contiguous United States (COThetaS): Mizukami et al. (2017) and Tsai et al. (2021). Mizukami et al. (2017) used an MPR based approach (MPR‐flex) which uses TFs chosen from literature. They concluded that TFs with global parameters lead to improve spatial fields, that there is still a large gap in performance between a global parameter set and individual basin calibration for the chosen TFs, and “though not trivial” different forms of TFs should be evaluated. Overall, Mizukami et al. (2017) shows the advantage of the MPR approach, while being limited by TFs from literature that were not developed for large‐scale modeling systems. Especially since VIC uses multiple conceptual parameters, which limits the use of literature‐based TFs, this study would have benefited from the FSO approach. Tsai et al. (2021) developed a deep learning approach for parameter estimation called differentiable parameter learning (dPL). dPL estimates parameters by optimizing a neural network that generates model parameters. To optimize this neural network, it is necessary to either have a fully differentiable hydrological model or to use a surrogate neural network instead of the hydrological model. They show that the dPL approach improves the discharge prediction results of Mizukami et al. (2017) from a median NSE of 0.32–0.44. Tsai et al. (2021) argue that dPL produces better generalizability and physical coherence of the derived parameters based on the fact that dPL uses dynamic and static catchment attributes as inputs and improves soil moisture simulation compared to a model optimization using calibration with the SCE‐UA algorithm. dPL is dependent on a well‐performing surrogate model, or on re‐writing the existing model to make it differentiable. By comparison, we demonstrate that FSO can identify TFs that can be applied to any model without changing them. While MPR‐flex and dPL were only tested with the VIC model in a gauged setting, they show the current state of approaches for deriving model parameters based on physiographic properties of catchment and highlight the complexity of this task.

This study has some limitations. First, while Zink et al. (2017) only used data from the calibration basins to derive the parameter sets, we also used 20 sampled validation basins to find the best FSO run. Since we applied FSO multiple times and were also interested in the variance of FSO performance, comparing them with data outside the calibration basins was necessary. Nevertheless, these sampled validation results showed comparable performance to the calibration and consequently did not strongly influence our choice of the best run. Another limitation is the fact that we only use 5 FSO runs, which is due to very practical reasons: the resources of the cluster that we used for computation are limited. However, we do believe that the 5 runs provide a useful estimate of the TF variability. Another limitation is the fact that we compare the performance of one FSO derived parameter set with the median performance of 100 parameter sets derived by Zink et al. (2017). This certainly shows the general performance of the original mHM TFs, but will also lead to a reduction in variance in the performance of Zink et al. (2017) due to aggregation. Therefore, from the presented results we cannot conclude that there is one specific Zink et al. (2017) parameter set that performs equal, better, or worse than the FSO parameter set, but shows that their performance is comparable.

Some limitations and opportunities for development also exist concerning the FSO method. The dynamic parts of the loss function, weights of the objective function, and the TF length‐dependent penalty, potentially increase loss function complexity that could affect convergence during optimization. While these dynamic adaptions of the loss are based on essential aims of the FSO application, it is still unclear if they lead to relevant improvements or only to additional complexity. Another issue related to the search space complexity is also the autoencoder performance. Currently, there are prediction uncertainties in more complex TFs generated from function space, that potentially also lead to a search space that is not easily searchable. A better‐performing autoencoder could lead to a search space that can also enhance optimization. Finally, the current amount of calibration runs (up to 5,000) might limit the performance of FSO, since estimating TFs is a complex multi‐modal optimization problem. Given a certain timeframe that is available for such a study, this could potentially be improved by either increasing the number of iterations while decreasing the run time of a single hydrological model iteration or by implementing an optimization algorithm that allows parallel evaluation of multiple candidate solutions instead of the currently used serially progressing algorithms.

## 6 Summary and Conclusions

In this study, we presented the first large‐scale application of FSO for automatic TF estimation of a complex distributed hydrological model. We assessed the performance variability of the FSO method by applying it 5 times, which resulted in 3 nearly equally well‐performing sets of TFs and two with slightly lower performance. The final selected TFs resulted in predictions in 222 validation basins with a median NSE of 0.68. The performance was equal to the median performance of 100 predictions of the benchmark study of Zink et al. (2017).

Overall, this study is a proof‐of‐concept where we showed that FSO is able to produce state‐of‐the‐art results when applied to a complex distributed model, but more work is needed to derive physically meaningful parameter fields. We see some important aspects that have the potential to greatly improve TF estimation. First, this includes a careful selection of calibration basins, ideally with a wide range of physiographic characteristics but a low internal variance of these characteristics. Second, it is important to include further constraints during optimization in form of additional boundary conditions, for example, simultaneously optimizing discharge and evapotranspiration, to further constrain the optimization and allow for physically sound parameter fields. Finally, an extension of available physiographic properties available for FSO will potentially allow finding a better representation for a larger number of model parameters.

The multiscale parameter regionalization technique (MPR), which uses TFs, was described as a promising way forward for global hydrological and Land Surface models. With FSO we now have a method that can automatically estimate these TFs for any model, which will make it possible to apply global hyperresolution models “everywhere” in the future.

## Figures and Tables

**Figure A1:**
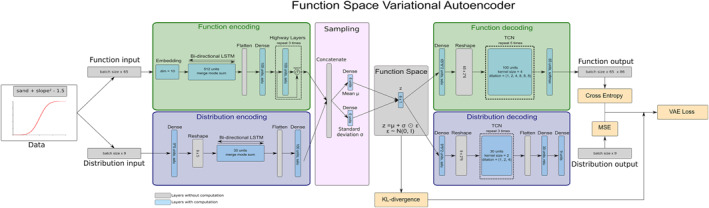
Function Space variational autoencoder architecture. Starting from the left: the data is separated into the function strings (Function input) and function quantiles (Distribution inputs). Each input has its own encoder network (Function encoding, Distribution encoding). The encoded ionputs are concatenated and used as basis for the mean and standard deviation sampling in the Sampling layer. These are then used to generate the normal distributed Function Space. The Function Space (6 dim vector) is used by the Function decoding and Distribution decoding networks to generate the Function and Distribution outputs, which are reconstructions of the inputs. The loss is computed with the sum of KL‐divergence of the Function Space to a standard normal distribution, the cross‐entropy of function string reconstruction and the MSE of function quantile reconstruction.

**Figure D1:**
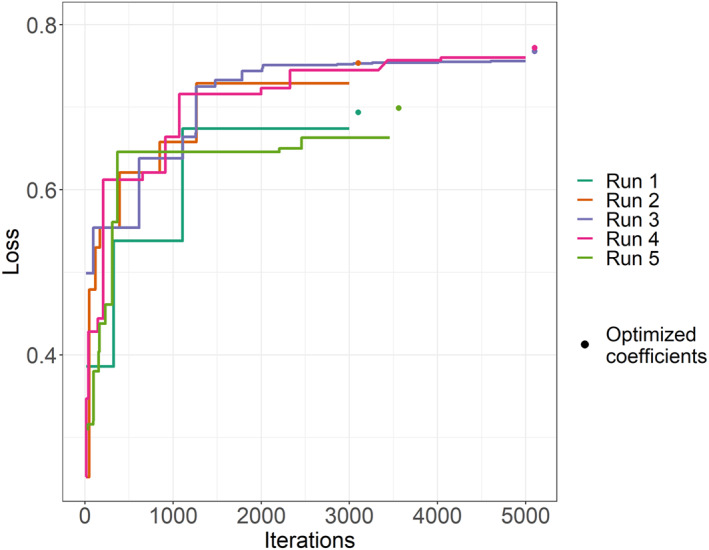
Overview of loss per iteration of all Function Space Optimization optimization runs during calibration. Only values above 0 are shown to increase readability. The optimization was applied for a minimum of 2,000 iterations and for a maximum of 5,000 iterations. It is stopped if after the minimum required iterations there is no further improvement for 1,000 iterations. After reaching one of these stopping criteria, only the numeric coefficients of the tranfer functions were optimized for 100 iterations, leading to additional improvement at the end of each run. This additional improvement is depicted as a point at the end of each run.

## Data Availability

The study data that was used in this study was previously made freely accessible by Zink et al. (2017) under Creative Commons license at www.ufz.de/index.php?en=41160. The mHM is available in Samaniego et al. (2019). The scripts used to produce all results of this study are available under Feigl et al. (2022), which also use functions from Feigl (2022).

## Appendix A FSO VAE Architecture & VAE Training

The new encoder network of the VAE consists of a word embedding layer (Mikolov et al., 2013), bidirectional long short‐term memory layers (Hochreiter & Schmidhuber, 1997), highway layers (Srivastava et al., 2015) using feedforward neural networks (FNN) (White & Rosenblatt, 1963) and the SELU (scaled exponential unit) activation function (Klambauer et al., 2017). The new VAE decoder consists of Temporal Convolutional Network layers (Bai et al., 2018) and a FNN using a softmax activation function. The function space is chosen to be 6‐dimensional and normal distributed. A detailed depiction of the VAE architecture is shown in Figure A1.

## Appendix B FSO Setup Details

### B1 Context Free Grammar (CFG) Used to Generate Training Data

$$\begin{array}{ccc} eq & = & eq\;op\;eq\;|\;eq\;op\;numeric\;|\;eq\;op\;\mathrm{var}\;\;|\;eq\;op\;\left(eq\right)\;|\;\mathrm{var}\;\;|\\ & & f\left(\mathrm{var}\;\right)\;|\;f\left(eq\right)\;|\;{\left(eq\right)}^{\wedge }\left(pm\;numeric\right)\;|\;numeric\\ op & = & +\;|\;-\;|\;*\;|\;/\\ \text{pm} & = & +\;|\;-\\ f & = & \exp\;\;|\;log10\;|\;\log\;\;|\;\sin\;\;|\;\cos\;\;|\;\tan\;\;|\;asin\;|\;acos\;|\;atan\;|\;\tanh\;\;|\\ & & \cosh\;\;|\;\sinh\;\;|\;sqrt\;|\;abs\\ \mathrm{var}\; & = & bd\;|\;sand\;|\;clay\;|\;slope\;|\;aspect\;|\;dem\;|\;ThetaS\;|\;K_{S}\;|\;vGenu_{n}\\ numeric & = & 0.05\;|\;0.1\;|\;\ldots \;|\;2.95\;|\;3.0 \end{array}$$

### B2 Scaling Bounds

The parameter bounds for the two estimated parameter were chosen to be *K*_*S*_ = [1.1, 1,000] and *FieldCap* = [0.01, 0.55]. The scaling bounds for the physiographic catchment properties is shown in Table B1.

**Table B1:** Scaling Bounds for Scaling Physiographic Catchment Properties and mHM Parameters to [0,1]

Physiographic property	Bounds
Slope	[0, 90]
Aspect	[0, 360]
*bd*	[0, 2.3]
Sand	[0, 100]
Clay	[0, 100]
*dem*	[0, 4,000]
$vGenu_{n}$ ^a^	[1, 2]
*ThetaS* ^a^	[0.24, 0.51]

^a^ Estimated from default mHM parameter values.

The bounds for scaling TF outputs to the parameter range are automatically estimated using the FSO VAE training data. The lower bound is the *median* 10% *quantile* + 3 × *mad*(10% *quantile*) and the upper bound is the *median* 90% *quantile* + 3 × *mad*(90% *quantile*), where *mad* denotes the median absolute deviation. Resulting in the ranges *K*_*S*_ = [−5.606, 8.481] and *FieldCap* = [−5.498, 8.50].

### B3 Hyperparameter of Optimization Algorithms:

The used DDS and SCE algorithms are our own *R* implementation and the GA algorithm was taken from the *GA* *R* package (Scrucca, 2013).

- DDS: *r* = 0.2, *iterations* = 5,000
- SCE: *ncomplex* = 5, *pointscomplex* = 50, *pointssimplex* = 10, *cce*.*iter* = 5, *elitism* = 1, *initsample* = *”latin”*, *iterations* = 5,000
- GA: *pop*_*size* = 10, *iterations* = 100

### B4 VAE Training

Each of the two VAEs (one for KSat and one for FieldCap TFs) is trained using 36,213,600 unique training samples and validated using 9,053,400 unique validation samples generated from the CFG. The VAEs are trained using the Adam optimization algorithm with a learning rate of 0.001. The batch size was chosen to be 250. Due to the extremely large number of samples (1 epoch equals 144,854 training steps) compared to the complexity and variety of data, the VAEs converged in only a few epochs. The KSat VAE hat the highest performance after 12 epochs and the FieldCap VAe hat the highest performance after 11 epochs.

## Appendix C Performance Metrics Overview

(C1)
$$NSE\left(Q_{\mathrm{obs}},Q_{\mathrm{sim}}\right)=1-\frac{\sum_{t}Q_{\mathrm{sim}}^{t}-Q_{\mathrm{obs}}^{t}}{\sum_{t}Q_{\mathrm{obs}}^{t}-{\bar{Q}}_{\mathrm{obs}}}$$

(C2)
$$\log NSE\left(Q_{\mathrm{obs}},Q_{\mathrm{sim}}\right)=1-\frac{\sum_{t}\log\;Q_{\mathrm{sim}}^{t}-\log\;Q_{\mathrm{obs}}^{t}}{\sum_{t}\log\;Q_{\mathrm{obs}}^{t}-\text{lo}\bar{\text{g}}\;Q_{\mathrm{obs}}}$$

(C3)
$$\begin{array}{ccc} KGE\left(Q_{\mathrm{obs}},Q_{\mathrm{sim}}\right) & = & 1-ED\\ ED & = & \sqrt{{\left(\left(r-1\right)\right)}^{2}+{\left(\left(\alpha -1\right)\right)}^{2}+{\left(\left(\beta -1\right)\right)}^{2}}\\ r & = & \text{Pearson}\;\text{correlation}\;\text{coefficient}\\ \alpha & = & \sigma_{\mathrm{sim}}/\sigma_{\mathrm{obs}}\\ \beta & = & \mu_{\mathrm{sim}}/\mu_{\mathrm{obs}} \end{array}$$

(C4)
$$PBIAS\left(Q_{\mathrm{obs}},Q_{\mathrm{sim}}\right)=100\times \left(\sum_{t}Q_{\mathrm{sim}}^{t}-Q_{\mathrm{obs}}^{t}\right)/\sum_{t}Q_{\mathrm{obs}}^{t}$$

## Appendix D Optimization Progress

Figure D1 shows the progression during optimization of all 5 FSO runs. The DDS optimizer (run 5) converges rapidly to a comparatively low performance, similar to what we have already observed in our preliminary tests. The SCE optimizer (run 1–4) seems to have a much more continuous improvement, except for run 1, which shows a similar behavior to the DDS run. These optimization performances suggest that discarding run 1 and run 5 would be a reasonable choice.

The loss shown in this figure is the FSO optimization loss as described in Equation 4 consisting of the power mean of NSE and log NSE values of the 7 training basins and the length of the estimated TF. For the presented training setup it can be written as:

(D1)
$$f_{\mathrm{loss}}=-\frac{\sum_{i=1}^{7}w_{i}\Phi_{i}}{\sum_{i=1}^{7}w_{i}}-\lambda \left(TFs\right),\;\text{with}\;\text{each}\;w_{i}=1-\Phi_{i}$$

(D2)
$$\Phi_{i}={\left[\frac{1}{2}\left(NSE{\left(Q_{i,obs},Q_{i,sim}\right)}^{p}+\log\;NSE{\left(Q_{i,obs},Q_{i,sim}\right)}^{p}\right)\right]}^{\frac{1}{p}}$$

(D3)
$$\lambda \left(TFs\right)=\frac{1}{2}\sum_{m=1}^{2}0.001\;length\left(TF_{m}\right)$$

Here, Φ_*i*_ is the value of the objective function for basin *i*, *w*_*i*_ is the corresponding weight, *p* denotes the exponent of the *L*_*p*_ norm (Duckstein & Opricovic, 1980) that was chosen to be *p* = 6, and *λ*(*TFs*) the penalty for TF lengths.
